# Evaluation of Skinfolds Based Predictive Equations for Estimating Body Fat Percentage in Older Adults: A Comparison Using Digital Adipometer (Lipowise) and Dual‐Energy X‐Ray Absorptiometry (DXA)

**DOI:** 10.1111/cob.70063

**Published:** 2025-12-10

**Authors:** Gabriela Benatti de Oliveira, Lara Vilar Fernandes, Teresa F. Amaral, Ana Carolina Junqueira Vasques, Ligiana Pires Corona

**Affiliations:** ^1^ Faculty of Medical Sciences State University of Campinas Campinas Brazil; ^2^ Faculty of Applied Sciences State University of Campinas Limeira São Paulo Brazil; ^3^ Faculty of Nutrition and Food Sciences UPorto University of Porto Porto Portugal

**Keywords:** adipose tissue, anthropometry, body composition, older adults, skinfold thickness

## Abstract

Accurate assessment of body fat percentage (%BF) is essential for predicting health risks in older adults, as age‐related changes affect fat distribution and measurement accuracy. While dual‐energy x‐ray absorptiometry (DXA) is a standard reference, it is costly and less accessible. This pilot study evaluated the validity of four skinfold equations using a digital calliper, with DXA as the reference to estimate %BF in older adults. A cross‐sectional study included 149 participants (28 men and 121 women) aged 60 to 86 years. The Lean, Durnin‐Womersley, and Visser equations (using 2 and 4 skinfolds) were analysed to estimate %BF. Statistical analyses were performed using SPSS (v.29), including Student's t‐test, Mann–Whitney, Cohen's Kappa, and Bland–Altman. Skinfold equations differed significantly from DXA‐derived %BF. In women, DXA values were lower than Lean but higher than Durnin‐Womersley; in men, DXA showed lower %BF than Lean and higher than others. Agreement with DXA was moderate (Kappa 0.54–0.59). Durnin‐Womersley underestimated %BF, especially in men (11.5%); Lean slightly overestimated (1.3%). Visser showed minimal bias—slight overestimation in women (0.3%, 0.4%) and underestimation in men (3.7%, 4.3%). Age‐related physiological factors may impact equation accuracy. Discrepancies between DXA and skinfold equations highlight the need for age‐specific prediction models for older adults.

## 1 Introduction

Aging causes significant changes in body composition, including muscle mass loss, increased body fat, and fat infiltration into muscles, all of which are factors associated with functional decline in older adults [1, 2].

Additionally, there is a redistribution of body fat, with an increase in abdominal visceral fat and a reduction in lower limb subcutaneous fat, which occurs differently between sexes, particularly after menopause. These changes in body composition can be influenced by both physiological factors and lifestyle. Aging is associated with a reduction in anabolic response, systemic inflammation, and mitochondrial dysfunction, all of which impair muscle regeneration and promote the accumulation of visceral and ectopic fat [3, 4, 5, 6].

Obesity in older adults is linked to a greater risk of adverse outcomes such as falls, disability, premature mortality, and long‐term care admissions. These risks are exacerbated by age‐related declines in metabolism and physical activity and contribute to the emergence of aging‐related phenotypes like sarcopenic obesity and frailty [7, 8].

Body composition assessment can be performed using various methods. While BMI is widely used, it has limitations, as it does not differentiate between lean mass and fat mass, and is influenced by height reduction with aging. Re precise techniques include dual‐energy x‐ray absorptiometry (DXA), magnetic resonance imaging (MRI), and computed tomography (CT). While CT and MRI are considered gold standards, their high costs, longer processing times, and—particularly for CT—radiation exposure, limit their use in routine clinical practice. In contrast, DXA has gained popularity for offering accurate and relatively accessible measurements of both total and regional body composition [8, 9].

Other alternatives include bioelectrical impedance analysis (BIA) and air displacement plethysmography, which provide practical estimates of body composition. Simple anthropometric measures, such as waist circumference, are useful for identifying central adiposity, which is associated with cardiometabolic risks [6, 8, 10].

Anthropometry is widely used to estimate body fat due to its low cost and ease of application, making it popular in both research and clinical settings. Skinfold (SF) thickness measurements are a key anthropometric method for estimating subcutaneous fat in specific regions. However, challenges such as the redistribution of subcutaneous fat, the choice of equations, and measurement techniques can affect accuracy, particularly in older adults. Equations based on measurements like weight, height, and waist and hip circumference are inexpensive, non‐invasive, and associated with smaller errors compared to those using SF thickness. In older adults, SF becomes more prone to errors due to morphological changes, including reduced skin elasticity, redistribution of body fat, and loss of muscle and bone mass [11, 12, 13].

However, applying anthropometric equations validated in specific populations to others requires careful evaluation. Factors such as age, sex, ethnicity, and body fat distribution must be considered when selecting the best equation. The population being assessed should ideally resemble the sample used for equation validation [14, 15]. While accurate body fat percentage (%BF) diagnosis is crucial, studies validating these equations for older adults remain limited [11, 16, 17].

Several factors influence the accuracy of these equations, including age, health conditions, ethnicity, and the reference method. For instance, Deurenberg et al. [18] found that equations based on general adult populations overestimated fat‐free mass by 6–7 kg in older adults. This error arises from differences in body composition, such as bone mineral content and total body water, between younger and older adults [19]. The lack of research concerning the study of the validity of these equations for older adults highlights a significant gap in the literature, emphasising the need for further studies to improve their precision and applicability.

As this investigation was conducted with a relatively small sample size and convenience‐based recruitment, it should be regarded as a pilot study, that aims to evaluate the validity of four anthropometric equations to estimate %BF in Brazilian older adults, using the Lipowise digital calliper and comparing the results with DXA, the reference method. The findings of this pilot study are expected to provide valuable information that will help select more accurate equations for estimating %BF, thereby assisting clinical practice in monitoring the health of older adults.

## 2 Material and Methods

### 2.1 Design and Subjects

This research is an observational study, of the cross‐sectional type, that examined information gathered from 149 older adults (≥ 60 years) residing in Campinas, Brazil. Participants were drawn from three distinct groups: (1) 73 enrollees in the UniversIDADE Program at the State University of Campinas (UNICAMP), an initiative offering courses and activities for individuals in the pre‐retirement, retirement, and post‐retirement phases of life; (2) UNICAMP staff members; and (3) patients receiving care at the Geriatric Outpatient Clinic of the UNICAMP Clinics Hospital. The UNICAMP Ethics Committee gave its approval to this study (Approval Number: 51443321.0.0000.5404). Each participant provided written informed consent.

The study included individuals aged 60 and above, living in Campinas or nearby communities, possessing the neurological and cognitive capacity to respond to questionnaires, and demonstrating sufficient mobility for physical evaluations. Individuals receiving home healthcare, undergoing chemotherapy, or presenting conditions known to substantially affect body composition (e.g., chronic obstructive pulmonary disease (COPD), chronic kidney disease requiring dialysis, Parkinson's disease, congestive heart failure), or those with HIV were not eligible to participate. Further information regarding participant recruitment and sample characteristics has been reported elsewhere [20, 21].

### 2.2 Dual Energy X‐Ray Absorptiometry

Dual‐energy x‐ray absorptiometry (DXA) is considered the reference method for assessing body composition, providing precise measurements of bone mineral density, bone mineral content, fat mass, and lean mass [22]. The examination is quick, lasting between 5 and 20 min [22], and exposes the patient to a low radiation dose (±10 μSv) [23].

In this study, the DXA device (Lunar, enCORE software, version 13.60; GE Healthcare) was used as the reference method for determining %BF.

The examination consisted of a whole‐body scan performed by an x‐ray source operating at two distinct energy levels, typically between 40–47 keV and 70–80 keV [23, 24]. The measurement of transmitted intensities from the two energy photons allows differentiation between body fat, bone mineral content, and lean soft tissue mass based on the varying radiological attenuation of the tissues [24].

To ensure data accuracy, the DXA equipment used in this study was calibrated daily before measurements, strictly following the manufacturer's guidelines [25, 26].

### 2.3 Lipowise

Lipowise is a patented digital skinfold calliper classified as a medical device that is connectable with iOS/Android APPs, that provides several features, including 48 equations applicable to 15 possible skinfolds to estimate body fat percentage. Lipowise applies a constant compression force of 10 gf/mm^2^ (error < 5%) with a resolution of 0.1 mm (error < 5%) at a sample rate of 100 Hz (100 values/s), which allows the profile to be traced and the tissue compressibility to be analysed [27, 28, 29].

The Lipowise PRO skinfold calliper from Lipowise, which originated from the Adipsmeter prototype (Lipotool, Portugal) [30], stands out in this range. Previous studies have shown that the Adipsmeter prototype was a very accurate instrument [31] and that Lipowise PRO is a valid skinfold calliper for skinfold measurement compared to traditional dial skinfold callipers [32]. Compared to traditional skinfold callipers, Lipowise PRO offers an alternative method for skinfold assessment by automating data acquisition and analysis through a dedicated mobile application. Additionally, it enables the evaluation of tissue compressibility through its technology, which measures skinfold thickness at a rate of 100 data points per second and provides a graphical representation of tissue response to constant force exerted by the calliper [29, 30, 31, 32, 33]. In our data collection, three measurements were taken at each point of the skinfolds determined in our protocol, and the average was subsequently calculated, a value that was used in the estimation equations.

### 2.4 Equations to Estimate %BF

For the analyses proposed in this study, body fat percentage (%BF) estimation equations were selected based on several criteria, such as age, suitability for the study population, recurrence and frequency in the scientific literature, and the availability of components in our database. These components were obtained through skinfold measurements using the Lipowise digital skinfold calliper.

Four predictive equations were selected: Durnin and Womersley (1974), Lean et al. (1996), and Visser et al. (1994) (∑2 and ∑4 skinfolds) [34, 35, 36, 37]. Table 1 presents all specifications and components of each selected equation. In addition to skinfolds, waist circumference (WC) was used in the predictive equations and was measured at the smallest observable circumference.

**TABLE 1:** Predictive equations of %BF selected for the analyses and their main characteristics.

Authors	Age (years)	Equations	
Durnin & Womersley (1974) [34]	Women = 50–68 Men = 50–72	Men: BD = 1.1765–0.0744 × log10 (Σ4 skinfolds)	Women: BD = 1.1339–0.0645 × log10 (Σ4 skinfolds)
		Σ4 Skinfolds: sum tricipital brachii, subscapular, bicipital brachii, suprailiac	
Visser et al. (1994) [36]	60–87	BD = 1.0481 + 0.0186 × (sex) − 0.0300 × log10 (Σ2 skinfolds)	BD = 1.0688 + 0.0212 × (sex) − 0.0356 × log10 (Σ4 skinfolds)
		Σ2 Skinfolds: sum of bicipital brachii and tricipital brachii (mm) Σ4 Skinfolds: sum of bicipital brachii, tricipital brachii, subscapular, and suprailiac (mm) Sex: 0 = women; 1 = men	
Lean et al. (1996) [35]	18–65	Men: %BF = 0.353 × (WC) + 0.756 × (TS) + 0.235 × (age) − 26.4	
		Women: %BF = 0.232 × (WC) + 0.657 × (TS) + 0.215 × (age) − 5.5	
Brozek et al. (1963) [37]	—	% BF = [4.570/BD (kg/m^3^) − 4.142] × 100	

Abbreviations: %BF, percentage body fat; BD, body density; TS, tricipital skinfold, mm; WC, waist circumference, cm.

Results obtained from the Durnin & Womersley (1974) and Visser et al. (1994) equations are expressed as body density. Density values were converted into %BF using the Brozek equation [37]. According to the literature, the Brozek equation is considered more accurate than the Siri equation for converting body density to %BF in older adults, as it shows better agreement with DXA assessments [38].

### 2.5 Statistical Analyses

Statistical analysis was performed to evaluate the relationship between %BF measured by DXA and %BF estimated by prediction equations. Quantitative variables are presented as mean ± standard deviation − normal distribution or median (interquartile range) for variables that did not adhere to the normal distribution. Sex differences were analysed using independent samples t‐tests. Paired samples t‐tests were used to compare %BF values derived from DXA and the prediction equations.

To assess the agreement between DXA‐derived %BF and %BF from skinfolds and prediction equations, two methods were employed: relative agreement and Cohen's kappa with linear weighting. Participants were stratified into sex‐specific quartiles based on their %BF. Cohen's kappa interpretation followed established criteria: 0.00–0.20 (negligible), 0.21–0.40 (fair), 0.41–0.60 (moderate), 0.61–0.80 (substantial), and 0.81–1.00 (almost perfect) [39]. Relative agreement for each equation was calculated by determining the percentage of participants in each quartile.

Bland–Altman analysis was also used to visually assess agreement, with limits of agreement defined as the mean difference ± 2 standard deviations. All analyses were performed using SPSS version 29 (IBM Corp.), with a significance level of *p* < 0.05.

## 3 Results

The Characteristics of participants according to sex are depicted in Table 2. Significant differences (*p* < 0.001) were found between females and males for the variables: WC, biceps skinfold, triceps skinfold, suprailiac skinfold, and %BF assessed by DXA. The men showed higher values of WC, but the women showed higher values of skinfolds and %BF.

**TABLE 2:** Demographic and body composition characteristics of participants according to sex (*n* = 149).

Variables	Female (*n* = 121)	Male (*n* = 28)	*p*
Age (years)	69.4 (6.5)	70.8 ± 5.7	0.235
BMI (kg/m^2^)	28.3 (7.8)	28.9 ± 3.5	0.504
WC (cm)	84.5 (15.5)	100.7 (5.9)	< 0.001
Biceps skinfold (mm)	14.8 (7.0)	10.5 (3.6)	< 0.001
Triceps skinfold (mm)	21.0 ± 5.7	13.2 (4.9)	< 0.001
Suprailiac skinfold (mm)	16.0 (7.9)	14.3 (4.8)	< 0.001
Subscapular skinfold (mm)	20.9 ± 7.3	20.9 (8.6)	0.237
Body fat DXA (%)	41.9 ± 6.6	35.3 ± 5.6	< 0.001

*Note:* Data from variables that presented normal distribution are presented as mean ± standard deviation, and variables that did not adhere to normal distribution are presented as median (interquartile range).

Abbreviations: BMI, body mass index; DXA, dual‐energy x‐ray absorptiometry; WC, waist circumference.

Table 3 compares the %BF assessed by DXA and the %BF from skinfolds and equations (Lean, Durnin and Womersley, Visser 2 and 4 skinfolds). Women presented lower %BF values by DXA than %BF by the equation of Lean and, higher %BF values by DXA than %BF by the equation of Durnin and Womersley (*p* < 0.001). Men had a lower %BF value by DXA than %BF by the Lean equation (*p* = 0.023); presented higher %BF values by DXA than %BF by the Durnin and Womersley equation, Visser 2 and 4 skinfolds (*p* < 0.001).

**TABLE 3:** Comparison between the percentage of body fat assessed by dual‐energy x‐ray absorptiometry (DXA) and equations according to sex.

Equations	%Body fat equation	%Body fat DXA	*p*
Female (*n* = 121)			
Lean	43.5 ± 5.9	41.9 ± 6.6	< 0.001
Durnin and Womersley	36.3 ± 3.7		< 0.001
Visser 2 skinfold	41.9 ± 1.8		0.895
Visser 4 skinfold	41.5 ± 2.1		0.360
Male (*n* = 28)			
Lean	36.6 ± 5.4	35.3 ± 5.6	0.023
Durnin and Womersley	23.8 ± 3.7		< 0.001
Visser 2 skinfold	31.6 ± 1.6		< 0.001
Visser 4 skinfold	31.1 ± 1.8		< 0.001

*Note:* Data are presented as mean ± standard deviation; (Lean equation − Wilcoxon test for both sexes; Visser 2 skinfolds equation—Wilcoxon test for women and paired t‐test for men; Visser 4 skinfolds equation—paired t‐test for both sexes; Durnin and Womersley equation—paired t‐test for both sexes).

Table 4 shows the agreement between the %BF assessed by DXA and equations (Lean, Durnin and Womersley, Visser 2 and 4 skinfolds). The equations presented moderate agreement (Cohen's Kappa = 0.54 − 0.59) with the %BF assessed by DXA.

**TABLE 4:** Agreement between the percentage of body fat assessed by Dual‐energy x‐ray absorptiometry (DXA) and equations (*n* = 149).

% Body fat	Weighted Cohen's Kappa	95% limits of agreement
DXA		
Lean	0.57	0.49–0.66
Durnin and Womersley	0.58	0.50–0.67
Visser 2 skinfold	0.56	0.47–0.65
Visser 4 skinfold	0.58	0.50–0.67

**FIGURE 1:**
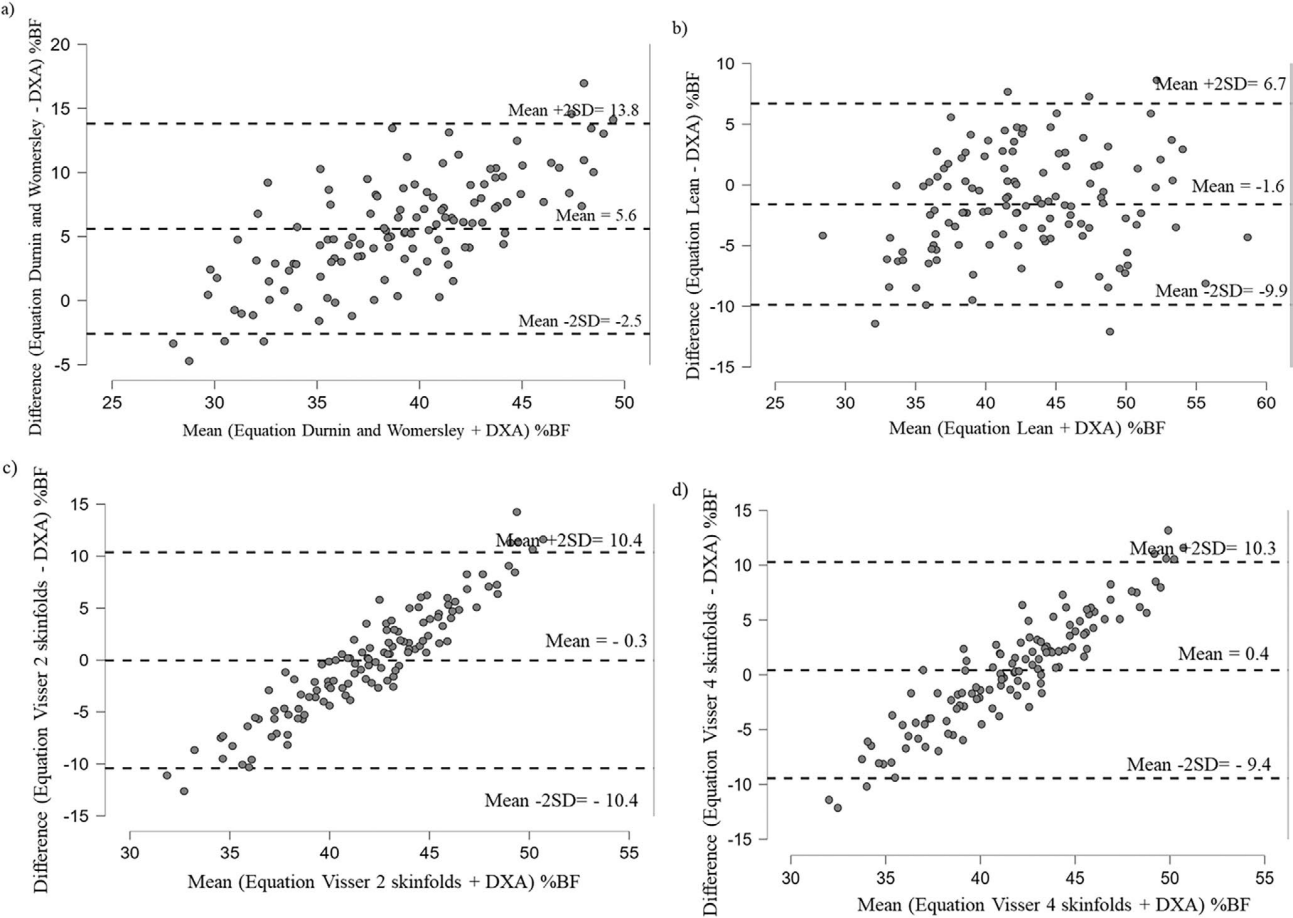
Agreement between the percentage of body fat (%BF) assessed by Dual‐energy x‐ray absorptiometry (DXA) and equations in women (*n* = 121). (a) Agreement between the %BF by DXA and the Equation of Durnin and Womersley; (b) Agreement between the %BF by DXA and the Equation of Lean; (c) Agreement between the %BF by DXA and the Equation of Visser 2 skinfolds; (d) Agreement between the %BF by DXA and the Equation of Visser 4 skinfolds.

Figure 1 shows the Bland–Altman agreement analysis between the %BF assessed by DXA and the %BF from skinfolds and equations for females. Compared to the %BF by DXA, the Durnin and Womersley equation underestimated the adiposity by 5.6 %BF (Figure 1a); the Lean equation overestimated adiposity by 1.6% (Figure 1b); the Visser 2 skinfolds and Visser 4 skinfolds overestimated adiposity by only 0.3% and 0.4%, respectively (Figure 1c,d), also show a trend in the distribution of points. This inclination may indicate a proportional bias, suggesting that the differences between the methods vary depending on the %BF values. This pattern hypothetically highlights the need for caution when using these equations across different %BF ranges.

**FIGURE 2:**
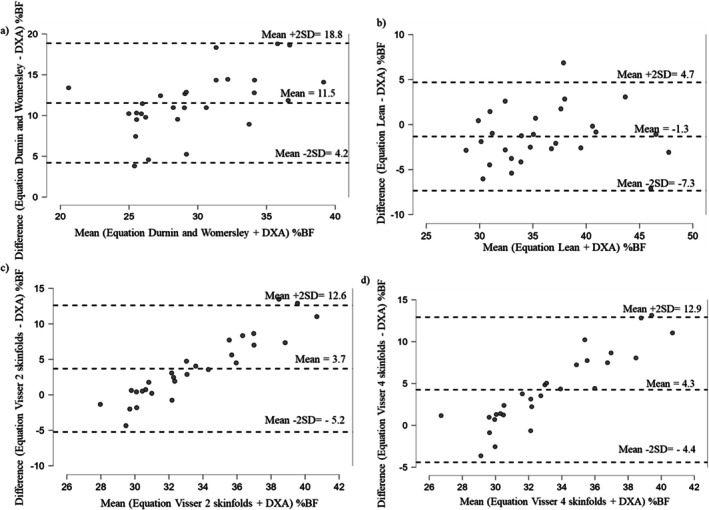
Agreement between the percentage of body fat (%BF) assessed by Dual‐energy x‐ray absorptiometry (DXA) and %BF from skinfolds and equations in men (*n* = 28). (a) Agreement between the %BF by DXA and the Equation of Durnin and Womersley; (b) Agreement between the %BF by DXA and the Equation of Lean; (c) Agreement between the %BF by DXA and the Equation of Visser 2 skinfolds; (d) Agreement between the %BF by DXA and the Equation of Visser 4 skinfolds.

Figure 2 shows the Bland–Altman agreement analysis between the %BF assessed by DXA and the equations for males. Compared to the %BF by DXA, the Durnin and Womersley equation underestimated the adiposity by 11.5 %BF (Figure 2a); the Lean equation overestimated adiposity by 1.3% (Figure 2b); the Visser 2 skinfolds and Visser 4 skinfolds underestimated adiposity by 3.7% and 4.3%, respectively (Figure 2c,d).

## 4 Discussion

The findings of this study provide important insights into the selection of more accurate equations for estimating %BF, which can enhance clinical practice in the monitoring and management of the health of older adults. Additionally, we observed different levels of agreement between the %BF measured by DXA and the estimates generated by the %BF from skinfolds and Lean, Durnin‐Womersley, and Visser equations, with notable variations between sexes.

Similar results were found in previous studies. For instance, a study conducted with adult women of different ethnicities (African American and Caucasian) demonstrated that skinfolds at the triceps, thigh, and supra‐iliac sites showed a higher agreement with DXA measurements. Moreover, the authors highlighted that some equations are more accurate when adjusted for racial specificity, emphasising that individual characteristics can influence the effectiveness of predictive equations [40].

A study with older adults assessed the %BF from skinfolds and Woolcott equation and its relationship with DXA in estimating %BF. Despite the absence of significant differences, high errors compromised its applicability, especially in short women. The study also indicated that BIA showed good agreement with DXA in taller women (> 145 cm), while anthropometric equations exhibited greater variability in shorter women, highlighting the importance of considering individual characteristics when choosing a method to estimate %BF [41].

Differences in body composition were also evident in studies investigating the relationship between %BF, age, and BMI. A study with healthy adults indicated that %BF increases with age in both sexes, while lean mass decreases in men and remains stable in women. These physiological changes may compromise the accuracy of predictive equations that do not account for these variations [42].

Additionally, a Brazilian study developed and validated a new equation to estimate %BF in older women, based on BMI and age. The %BF from skinfolds and this equation strongly agrees with x%BF DXA values, suggesting that models specific to older populations may yield more accurate estimates [43, 44].

Another study investigated body fat prediction models that included variables such as age, BMI, anthropometric measures, and skinfolds. These models exhibited mean errors lower than 0.10% body fat in both sexes, whereas pre‐existing methods, such as the Durnin‐Womersley and Woolcott equations, either overestimated or underestimated %BF significantly [45].

Present study also corroborates previous findings regarding the influence of individual characteristics on the accuracy of predictive equations. The study showed significant variations in results when comparing different equations with DXA, emphasising that no equation is universally applicable without considering population‐specific characteristics [46].

As in this study, our findings indicate that the accuracy of %BF from skinfolds and predictive equations varies across groups. In the case of adolescents with obesity, some equations overestimated or underestimated %BF. Similarly, our results show that the %BF from skinfolds and the Durnin‐Womersley equation underestimates %BF, particularly in men, while the %BF from skinfolds and the Lean equation tends to slightly overestimate. This pattern suggests that the choice of equation should consider characteristics such as age, fat distribution, and body composition. Thus, the results from this and other studies reinforce that there is no single ideal equation for all population groups. Factors such as aging, fat distribution, and body composition affect the accuracy of equations, highlighting the need for continuous validation of models in different populations.

Some limitations of our study should be highlighted. First, due to the relatively small sample size and convenience‐based recruitment, this pilot study provides preliminary evidence on the validity of skinfold‐based predictive equations for estimating %BF in older adults. Consequently, the results cannot be generalised to other ethnicities, as the sample consisted of older Brazilian adults. However, it is worth emphasising that miscegenation in Brazil follows a north–south genetic gradient, with a greater African and Amerindian influence in the north and an increasing European contribution toward the south. This process has resulted in significant regional differences, reflected in distinct body compositions shaped by genetic and historical regional factors [47]. Additionally, since this was a, predominantly female convenience sample, extrapolating the findings to men should be done cautiously. It is also important to note that we used only a few predictive equations to estimate %BF, which may have excluded potentially more accurate methods, such as MRI and CT. Finally, although DXA is widely used as a reference method for body composition assessment, it is not considered a gold standard.

## 5 Conclusion

The results of this study highlight significant discrepancies between the values obtained from skinfolds and predictive equations and those obtained by DXA, emphasising the need for caution when interpreting results, especially in older populations. These differences reinforce the importance of considering factors such as age, sex, body fat distribution, and anthropometric characteristics when selecting a method for estimating %BF. Furthermore, our findings highlight the limitations of generalised equations and the need to develop and validate specific models for this population to ensure greater accuracy and clinical applicability. As this study was exploratory in nature, the results should be interpreted as preliminary evidence, providing a foundation for future research aimed at creating skinfold‐based equations tailored to the particularities of aging, considering both physiological and methodological factors that impact body composition assessment.

## Author Contributions

**Gabriela Benatti de Oliveira:** investigation, data curation, data analysis, and writing. **Lara Vilar Fernandes:** investigation, data curation, and data analysis. **Teresa F. Amaral:** writing. **Ana Carolina Junqueira Vasques:** supervision and writing. **Ligiana Pires Corona:** conceptualization, investigation, methodology, supervision, funding acquisition, and writing. All authors read and approved the final manuscript.

## Funding

This work was supported by the São Paulo Research Foundation (FAPESP) (Grant Numbers 2020/00944‐2 and 2021/01304‐0) and the Coordination for the Improvement of Higher Education Personnel (CAPES) (Funding Code 001). LPC thanks CNPq—Conselho Nacional de Desenvolvimento Científico e Tecnológico for her productivity grant (304838/2022‐5).

## Conflicts of Interest

The authors declare no conflicts of interest.

## Data Availability

Research data are not shared.
